# Neurophysiological In Vitro Model of Amyloid-β-Induced Deficits of Hippocampal LTP Involving Neuronal Adenosine A_2A_ Receptor Dysfunction Through CD73

**DOI:** 10.3390/cells15060510

**Published:** 2026-03-13

**Authors:** Francisco Q. Gonçalves, Henrique B. Silva, Ângelo R. Tomé, Paula Agostinho, Rodrigo A. Cunha, João P. Lopes

**Affiliations:** 1 Institute for Clinical and Biomedical Research (iCBR), Centre for Innovative Biomedicine and Biotechnology (CIBB), University of Coimbra, 3004-504 Coimbra, Portugal; 2 Department of Life Sciences, Faculty of Sciences and Technology, University of Coimbra, 3004-517 Coimbra, Portugal; 3 Faculty of Medicine, University of Coimbra, 3004-504 Coimbra, Portugal

**Keywords:** Aβ, hippocampus, neurophysiological model, LTP, adenosine, A_2A_ receptor, neurons

## Abstract

Amyloid-β peptides (Aβ) are considered a main culprit of Alzheimer’s disease (AD), leading to synaptic dysfunction and memory deficits. Although studies in animal models of AD converge to show alterations of synaptic plasticity, namely of long-term potentiation (LTP), the mechanisms through which Aβ affects synaptic function remain to be unveiled. In this study, we established experimental conditions showing that the acute exposure of mouse hippocampal slices to optimized concentrations of Aβ impaired short-term (PPF-paired-pulse facilitation) and long-term (LTP-long-term potentiation) plasticity without altering basal synaptic transmission. We observed that the elimination of extracellular adenosine with adenosine deaminase abrogated the impact of Aβ on synaptic plasticity, showing a mandatory involvement of extracellular adenosine in the neurophysiological effects of Aβ. Additionally, inhibiting adenosine receptor function with caffeine, as well as selectively blocking adenosine A_1_ receptors (A_1_R) with DPCPX, or adenosine A_2A_ receptor (A_2A_R) with either an antagonist SCH58261 or through knocking out A_2A_R, demonstrated that acute Aβ modified mouse hippocampal PPF via A_1_R and LTP through A_2A_R. Furthermore, the use of slices from mice bearing forebrain-neuron A_2A_R deletion, along with the application of α,β-methylene ADP, a CD73 inhibitor, confirmed that the neurophysiological actions of Aβ on hippocampal LTP occur selectively through the overfunction of neuronal A_2A_R via CD73-mediated formation of extracellular adenosine. Overall, the exploitation of a neurophysiological model of early AD, based on the acute administration of Aβ to hippocampal slices, confirmed the critical involvement of adenosine signaling in the impact of Aβ on synaptic plasticity.

## 1. Introduction

Amyloid peptides (Aβ) are the main constituents of senile plaques [1], one of the core neuropathological hallmarks of Alzheimer’s disease (AD) [2]. However, a primordial clinical feature of AD, the impairment of episodic memory [3], precedes the appearance of such plaques [4,5]. Indeed, at its onset, AD is proposed to be a synaptic disease [6,7], a contention confirmed by the loss of synapses and of synaptic markers, particularly in the hippocampus of early AD patients [8,9,10]. Likewise, we also previously reported a loss of hippocampal synapse markers in different animal models of AD involving Aβ accumulation [11,12,13]. This is associated with a synaptic dysfunction, typified by altered patterns of synaptic plasticity, namely long-term potentiation (LTP) and depression (LTD), considered the neurophysiological correlates of synaptic mechanisms underlying learning and memory [14]. Importantly, Aβ also affects astrocytic function [15,16] and bolsters neuroinflammation through the activation of microglia and astrocytes [17,18]. These glial alterations have been linked to changes in synaptic function [19,20,21]. Therefore, the need to devise novel candidate interventions with a solid rationale to manage early AD will require exploiting innovative models to disentangle if these new interventions directly control synaptic dysfunction or instead act on other mechanisms that indirectly affect synaptic function.

One example is the role of adenosine A_2A_ receptors (A_2A_Rs) in AD. A_2A_R polymorphisms are associated with the incidence of AD [22] and with alterations of hippocampal morphology in AD [23]. A_2A_Rs are upregulated in neurons and synapses in the hippocampus of AD patients [24,25] and of mouse models of AD [11,12,13,24], and their overactivation potentiates the onset of AD [26,27]. The pharmacological or genetic blockade of A_2A_R alleviates memory deficits in AD [11,12,13,24,28,29,30], an effect mimicked by caffeine [31,32,33,34,35], whose main mechanism of action in non-toxic doses is the antagonism of adenosine receptors [36,37]. However, the way by which A_2A_Rs control AD phenotypes is still unclear since these adenosine receptors regulate hippocampal synaptic plasticity [24,38,39,40], but also modulate astrocytic glutamate uptake [41] and neuroinflammation [42].

To directly investigate the control of synaptic plasticity, we now optimized a new in vitro model of Aβ-induced alteration of synaptic plasticity in hippocampal slices affording superior experimental reproducibility, mechanistic resolution, and translational screening potential. The exploitation of this model allowed us to demonstrate that CD73-dependent extracellular adenosine formation is mandatory for Aβ-induced LTP impairment, which specifically involves neuronal A_2A_Rs.

## 2. Materials and Methods

### 2.1. Animals

We used male and female C57bl\6j mice of 8–12 weeks of age, obtained from Charles River (Barcelona, Spain). Global A_2A_R knockout (gA_2A_RKO), forebrain-selective A_2A_RKO (fbA_2A_RKO), and CD73KO mice, as well as each individual control genotype (generically designated as wild type, WT), were generated and cross-bred as previously described [11,43]. Male and female mice were analyzed together, as previously done [26]. Mice were housed under controlled temperature (23 ± 2 °C), subject to a fixed 12 h light/dark cycle, with free access to food and water. Efforts were made to reduce the number of animals used and to minimize their stress and discomfort. All studies were conducted in accordance with the ARRIVE principles, with adherence to the guidelines of the European Community guidelines (Directive 2010/63/EU) and Portuguese law on animal care (1005/92), and approval from the Animal Care Committee of our Center (#300_2021/09112024) and the Portuguese Animal Ethics Committee (*Direcção Geral de Veterinária*, #0421/000/000/2021, 29 November 2021).

### 2.2. Drugs

Caffeine, α,β-methylene ADP (AOPCP), and adenosine deaminase (ADA) were sourced from Sigma (St. Louis, MO, USA); 8-cyclopentyl-1,3-dipropylxanthine (DPCPX) and 5-amino-7-(2-phenylethyl)-2-(2-furyl)-pyrazolo [4,3-e]-1,2,4-triazolo [1,5-c]pyrimidine (SCH58261) were obtained from Tocris (Bristol, UK). Drugs were used in supramaximal but selective concentrations: 100 μM AOPCP, 2 U/mL ADA, 100 nM DPCPX, 50 nM SCH58261. Caffeine was tested at a concentration of 50 μM (see [37]). AOPCP and caffeine were prepared in milliQ water to stock concentrations of 10 mM and 100 mM, respectively, and DPCPX and SCH58261 were prepared as 5 mM stock solutions in dimethylsulfoxide. The Aβ_1–42_ peptide fragment was purchased from Bachem (Bubendorf, Germany) and dissolved in milliQ water to obtain a solution mostly composed of Aβ oligomers [11,44] with a final concentration of 2.25 mg/mL.

### 2.3. Intracerebroventricular Injection of Aβ to Model Early AD

Mice were subjected to stereotaxic surgery for unilateral intracerebroventricular (icv) injection alternatively in the right or left hemisphere (dorsoventral: −2.00 mm; anteroposterior: −0.58 mm; lateral: +/−1.13 mm) of Aβ (single dose of 2 nmol of Aβ_1–42_ in 4 µL) or vehicle (water, which caused no behavioral or neurochemical effects) under anesthesia with avertin, as previously performed [11,30]. This dose of Aβ_1–42_ translates to levels of 5–30 pmol Aβ_1–42_ within the hippocampus, causing synaptic and reference memory dysfunction after 14 days without evidence of cellular damage [11], thus constituting a model of early AD.

### 2.4. Slice Preparation

Following decapitation, the brains were quickly removed and placed in ice-cold, oxygenated (95% O_2_, 5% CO_2_) artificial cerebrospinal fluid (ACSF; in mM: 124.0 NaCl, 4.4 KCl, 1.0 Na_2_HPO_4_, 25.0 NaHCO_3_, 2.0 CaCl_2_, 1.0 MgSO_4_, 10.0 glucose). The hippocampus was removed and 400 µm-thick slices were cut transversely to its long axis using a McIlwain tissue chopper (Brinkmann Instruments, Westbury, NY, USA). The hippocampal slices were placed in a holding chamber with oxygenated ACSF at 32 °C. The slices were allowed to recover for at least 1 h before being transferred to a submerged recording chamber. In the chamber, the slices rested on a nylon mesh and were superfused with oxygenated ACSF at 3 mL/min and kept at 30.5 °C.

### 2.5. Electrophysiological Recordings

Field excitatory postsynaptic potentials (fEPSP) were recorded as previously described [30,37] with the recording electrode, filled with 4 M NaCl (2–5 MΩ resistance), placed in the CA1 *stratum radiatum* targeting the distal dendrites of pyramidal neurons, and the stimulating bipolar concentric electrode placed over the afferent Schaffer fibers in the proximal CA1 *stratum radiatum*. Rectangular pulses of 0.1 ms were delivered every 20 s through a Digitimer pulse stimulator (DS3, Digitimer Ltd., Letchworth, UK). After amplification (ISO-80, World Precision Instruments Ltd., Hitchin, Hertfordshire, UK), the recordings were digitized (BNC-2110, National Instruments, Newbury, UK), averaged in groups of 3, and analyzed using the WinLTP version 2.10 software [45]. The intensity of stimulation was defined between 40 and 50% of maximal fEPSP response, determined on the basis of input/output (I/O) curves in which fEPSP slope was plotted vs. stimulus intensity. Alterations of synaptic transmission were quantified as the percentage modification of the average value of the fEPSP slope taken from 36 to 40 min after beginning the application of tested drugs (Aβ_1–42_) in relation to the average value during the 5 min before drug application. Paired-pulse facilitation (PPF), used as an indirect measure of presynaptic alterations, was elicited by stimulating twice with 25–200 ms inter-pulse intervals and quantified as the ratio between the slopes of the fEPSP elicited by the second and the first stimulus. Long-term potentiation (LTP) was induced by high-frequency stimulation (HFS—one train of 100 Hz for 1 s). LTP was quantified as the percentage change between two values: the average slope of the ten potentials taken between 51 and 60 min after LTP induction in relation to the values measured during the 10 min that preceded the HFS. The effect of tested drugs was assessed by comparing LTP magnitudes of untreated vs. slices from the same animal with the different treatments. Electrophysiological recordings and their analysis were performed in an unblinded manner.

### 2.6. Statistics

The values presented are mean ± S.E.M. of *n* experiments, corresponding to the number of animals. Alterations compared to baseline were estimated with a one-sample Student’s *t*-test, and the comparison of two experimental conditions was performed using Student’s *t*-test. Otherwise, statistical analysis was performed by one-way analysis of variance (ANOVA), followed by Tukey’s post hoc test. *p* < 0.05 was considered to represent a significant difference. Statistical analysis was performed using GraphPad Prism (ver. 8.0.1; GraphPad Software, San Diego, CA, USA) software.

## 3. Results

### 3.1. Establishment of an In Vitro Model of Altered Synaptic Function by Acute Aβ Exposure

**Figure 1:**
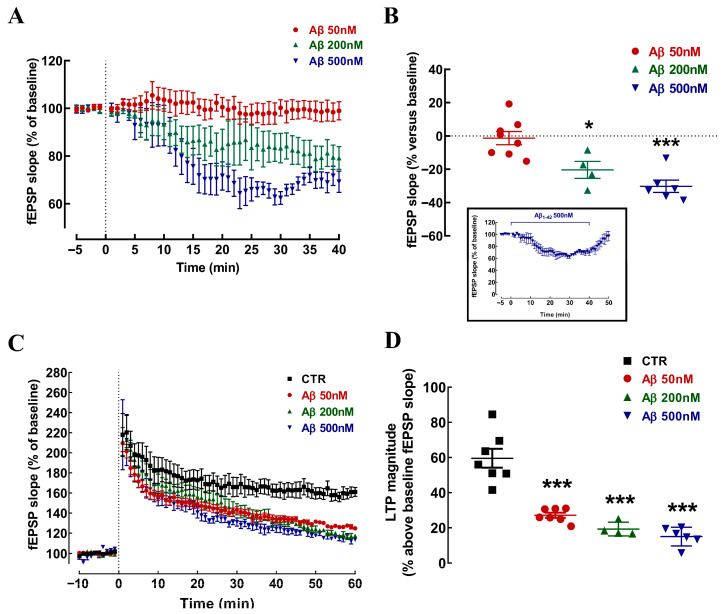
Oligomeric Aβ_1–42_ lowers synaptic transmission and impairs synaptic plasticity in a different concentration-dependent manner. Synaptic transmission was measured in Schaffer fibers–CA1 pyramid synapses by measuring the slope of field excitatory postsynaptic potentials (fEPSP). (**A**,**B**) Superfusion for 40 min with 200 and 500 nM but not with 50 nM of oligomeric Aβ_1–42_ (started as indicated by the vertical dashed line in (**A**)) decreased basal synaptic transmission. The insert in (**B**) is a time course experiment showing that the effect of 500 nM Aβ on synaptic transmission was reversible, since a 10 min washing period with ACSF (without Aβ) was sufficient to recover synaptic transmission after exposure to 500 nM Aβ. (**C**,**D**) The magnitude of long-term potentiation (LTP), triggered by a high-frequency train (HFS, 100 Hz for 1 s, indicated by the vertical dashed line in (**C**)) was similarly decreased by all tested concentrations of Aβ. Data are mean ± SEM of 4–8 (**A**,**B**) and 4–7 (**C**,**D**) experiments (number of animals tested); * *p* < 0.05, *** *p* < 0.001 compared to the absence of Aβ, depicted as baseline (horizontal dashed line in (**B**)), or control (CTR, (**D**)), one-sample Student’s *t* test (**B**) or one-way ANOVA followed by Tukey’s multiple comparison post hoc test (**D**).

We first aimed to establish an in vitro model of neurophysiological alterations triggered by Aβ in a representative neuronal circuit in the hippocampus. Considering the clear discrepancies between the concentrations and exposure times used in different previous studies addressing the neurophysiological effects of Aβ_1–42_ in hippocampal slices (e.g., [46,47,48,49,50]), we first opted for setting a fixed exposure period of 40 min and defining if and how different concentrations of Aβ affected synaptic transmission in Schaffer fibers–CA1 pyramid synapses of mouse hippocampal slices. Whilst 50 nM Aβ did not alter basal transmission (−1.34 ± 3.96% vs. baseline; n = 8; *p* > 0.05, one-sample *t* test), we observed a significant and incrementing decrease in basal synaptic transmission with the exposure to 200 nM Aβ (−20.39 ± 5.08% vs. baseline; n = 4; *p* < 0.05) and 500 nM Aβ (−30.24 ± 3.68% vs. baseline; n = 6; *p* < 0.001; one-sample *t* test for both concentrations) (Figure 1A,B). The impact of 500 nM Aβ on synaptic transmission was reversible, since a 10 min washing period with ACSF (without Aβ) was sufficient to recover synaptic transmission after exposure to 500 nM Aβ (n = 6; *p* < 0.05, one-sample *t* test) (insert in Figure 1B).

High-frequency stimulation (100 Hz for 1 s) elicited long-term potentiation (LTP), the magnitude of which was decreased by the different tested concentrations of Aβ (vs. control: −32.46 ± 4.63% for 50 nM Aβ, −40.32 ± 5.43% for 200 nM Aβ, and −44.53 ± 4.82% for 500 nM Aβ; n = 7, 4, and 6, respectively; *p* < 0.001 for all conditions, one-way ANOVA followed by Tukey’s multiple comparison *post hoc* test) (Figure 1C,D). The ability of 50 nM Aβ to selectively decrease LTP magnitude to an extent similar to higher Aβ concentrations without altering basal synaptic transmission prompted us to select the concentration of 50 nM of Aβ_1–42_ to establish the in vitro Aβ neurophysiological model of early AD.

**Figure 2:**
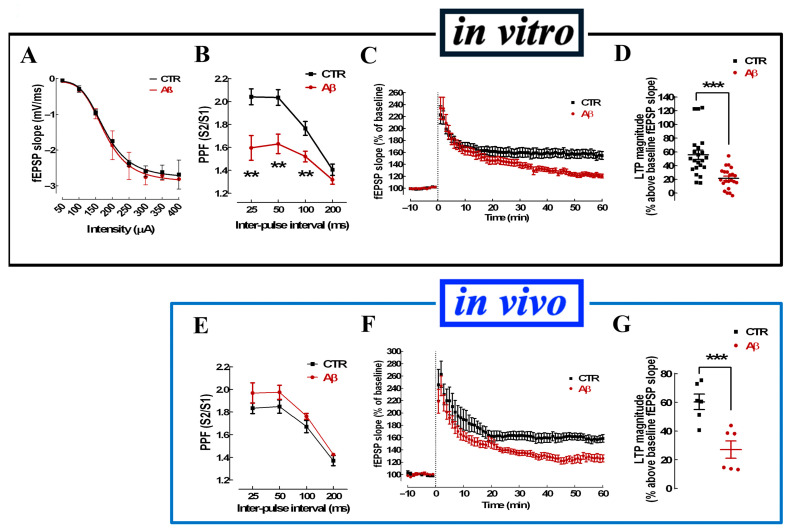
In vitro application of Aβ_1–42_ to slices alters hippocampal LTP in a manner similar to in vivo Aβ_1–42_ exposure. The input–output curves, measuring synaptic strength through the variation in fEPSP with increasing stimulation intensity, showed no differences upon direct application of Aβ (50 nM, 40 min) to mouse hippocampal slices (**A**). Conversely, acute Aβ significantly impaired paired-pulse facilitation (PPF), i.e., the ratio of the fEPSP slope of two consecutive pulses (S2/S1) at 25, 50, and 100 ms intervals (**B**), as well as LTP (**C**,**D**). In hippocampal slices from mice icv-injected with Aβ (14 days after a single injection of 2 nmol in 4 μL), although a similar impact was observed on LTP magnitude when comparing slices from vehicle- and Aβ-treated mice (**F**,**G**), no differences were observed on PPF (**E**). Data are mean ± SEM of 3–4 (**A**), 6–7 (**B**), 22 (**C**,**D**) 4–5 (**E**), and 6 (**F**,**G**) experiments (number of animals tested); ** *p* < 0.01, *** *p* < 0.001 compared to control, Student’s *t* test.

The 40 min exposure to 50 nM Aβ_1–42_ did not alter basal synaptic strength, as confirmed by the absence of significant differences in the input/output (I/O) curves with or without Aβ (Figure 2A). As shown in Figure 2B, Aβ (50 nM) decreased paired-pulse facilitation (PPF, a surrogate of presynaptic alterations) with inter-pulse intervals of 25 (1.60 ± 0.11 for Aβ vs. 2.04 ± 0.07 for control; n = 6-7; *p* < 0.01, Student’s *t* test), 50 (1.63 ± 0.09 for Aβ vs. 2.04 ± 0.07; n = 6–7; *p* < 0.01, Student’s *t* test) and 100 ms (1.52 ± 0.05 for Aβ vs. 1.77 ± 0.06; n = 6–7; *p* < 0.01, Student’s t test). Interestingly, in mice that were injected intracerebroventricularly with 2 nmol Aβ, PPF was similar in Aβ and H_2_O-injected mice (controls) (Figure 2E). This discrepancy between in vivo and in vitro exposure to Aβ was not observed for LTP. In fact, both in vitro (Figure 2C,D) and in vivo exposures to Aβ (Figure 2F,G) similarly decreased LTP magnitude (55.61 ± 6.78% over baseline without vs. 21.32 ± 3.21% with 50 nM Aβ acutely applied to slices; n = 22; *p* < 0.001; and 60.40 ± 5.37% over baseline in slices from control mice and 27.06 ± 5.96% in slices from Aβ-injected mice; n = 6; *p* < 0.001). Overall, these results indicate that the incubation of hippocampal slices for 40 min with Aβ (50 nM) selectively impairs synaptic plasticity without altering basal synaptic transmission.

### 3.2. The Neurophysiological Effects of Aβ Depend on the Presence of Extracellular Adenosine

**Figure 3:**
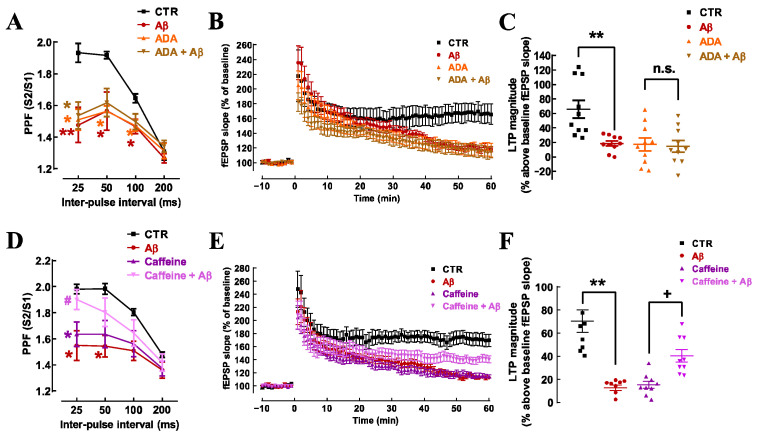
Extracellular adenosine signaling is crucial for the impairments of LTP and PPF by Aβ_1–42_. A previous superfusion with adenosine deaminase (ADA, 2 U/mL, which converts extracellular adenosine into its inactive metabolite inosine) occluded the effect of acute Aβ on PPF at all inter-pulse intervals (**A**) and on LTP magnitude (**B**,**C**), since Aβ had no additional effects in the presence of ADA on both forms of synaptic plasticity (**A**–**C**). Caffeine (50 μM, a non-selective antagonist of adenosine receptors) alone displayed a similar behavior on PPF (**D**) and LTP (**E**,**F**) as ADA alone, and reverted the impact of Aβ on both PPF (**D**) and LTP (**E**,**F**). Data are mean ± SEM of 4 (**A**), 10 (**B**,**C**), 5 (**D**), and 9 (**E**,**F**) experiments; * *p* < 0.05, ** *p* < 0.01 compared to control, # *p* < 0.05 compared to Aβ, n.s. *p* > 0.05 compared to ADA, + *p* < 0.05 compared to caffeine; one-way ANOVA followed by Tukey’s multiple comparison post hoc test.

Adenosine signaling operates an activity-dependent fine-tuning neuromodulation system, the blockade of which has previously been shown to attenuate the impact of Aβ on memory performance [11,12,32]. To evaluate if the effects of Aβ on synaptic plasticity depend on adenosine-mediated neuromodulation, we superfused hippocampal slices with adenosine deaminase (ADA, 2 U/mL), an enzyme that converts extracellular adenosine into inosine, an inactive metabolite in hippocampal slices. Similarly to what has been previously reported by our group [37], ADA decreased PPF (n = 4; Figure 3A). Notably, ADA completely occluded the effect of Aβ on PPF (n = 4; *p* > 0.05; Figure 3A). Moreover, ADA also occluded the impact of Aβ on LTP (Figure 3B,C). Thus, as previously reported by our group [37], ADA decreased LTP magnitude (−48.54 ± 12.32% decrease *vs*. control; n = 10; *p* < 0.01) and Aβ failed to cause any additional effect (*p* > 0.05 *vs*. effect of ADA alone) in the presence of ADA (−51.15 ± 12.32% vs. control; n = 10).

Since caffeine, at a concentration replicating that reached in the plasma upon moderate consumption of coffee, acts by blocking adenosine receptors to control information flow in hippocampal circuits [37], we next tested if caffeine (50 μM) also abrogated the impact of Aβ on synaptic plasticity in hippocampal slices. As for ADA, caffeine also decreased PPF inter-pulse ratio (Figure 3D) and impaired LTP magnitude (−55.21 ± 8.80% decrease vs. control; n = 9; *p* < 0.01; one-way ANOVA followed by Tukey’s multiple comparison post hoc test) (Figure 3E,F). Notably, caffeine reverted the effect of Aβ on PPF (*p* < 0.05 vs. Aβ without caffeine; Figure 3D) and on LTP (*p* < 0.05 vs. Aβ without caffeine; Figure 3E,F). This confirms that adenosine signaling is critically required for the ability of acute Aβ to depress LTP in hippocampal slices, as occurs for the control of memory deterioration upon chronic exposure to Aβ [11,12,32].

### 3.3. Aβ-Induced Impairment of PPF Involves A_1_R and Deterioration of LTP Involves A_2A_R

**Figure 4:**
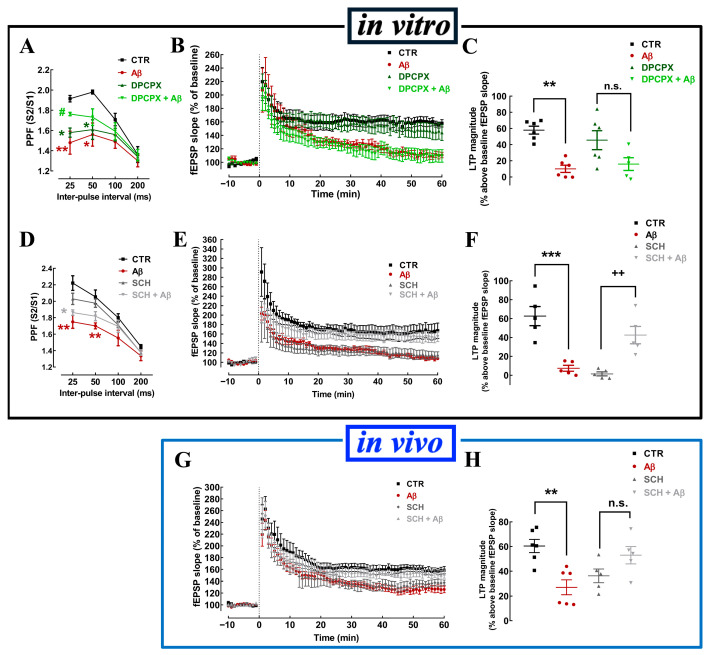
Aβ-induced depression of hippocampal PPF and LTP occurs via A_1_R and A_2A_R, respectively. The selective A_1_R antagonist DPCPX (100 nM) enhanced PPF (**A**), but did not modify LTP magnitude (**B**,**C**) induced with a high-frequency stimulation train (one train of 100 pulses of 1 Hz for 1 s, vertical dashed line in (**B**,**E**,**G**)) recorded in Schaffer fiber–CA1 pyramid synapses of mouse hippocampal slices directly exposed or not to Aβ. In contrast, the selective A_2A_R antagonist SCH58261 (SCH, 50 nM) did not significantly affect PPF deficits triggered by the superfusion with Aβ of naïve mouse hippocampal slices (**D**) but reverted the Aβ-induced decline of LTP magnitude (**E**,**F**). Notably, SCH58261 (50 nM) also reverted the depression of LTP magnitude in slices collected 14 days after in vivo icv injection of Aβ (**G**,**H**). Data are mean ± S.E.M. of 4 (**A**), 5–6 (**B**,**C**), 5 (**D**–**F**), and 5–6 experiments (**G**,**H**); * *p* < 0.05, ** *p* < 0.01, *** *p* < 0.001 compared to control, # *p* < 0.05 compared to Aβ (**A**), n.s. *p* > 0.05 compared to DPCPX (**C**) or SCH (**H**), ++ *p* < 0.01 compared to SCH (**F**); one-way ANOVA followed by Tukey’s multiple comparison post hoc test.

To reinforce the key role of adenosine signaling for the impact of Aβ on hippocampal synaptic plasticity, we investigated the contribution of the main adenosine receptors in the brain, namely A_1_ and A_2A_ receptors (A_1_R and A_2A_R), on the in vitro effects of Aβ in hippocampal slices. The selective A_1_R antagonist DPCPX (100 nM) significantly impaired PPF (Figure 4A) but was devoid of effect on LTP (57.88 ± 4.89% increase over baseline without and 45.49 ± 11.72% with DPCPX; n = 6; *p* > 0.05, Student’s *t* test) (Figure 4B,C). DPCPX abolished the impact of Aβ on PPF (n = 6; Figure 4A) but failed to prevent the impact of Aβ on LTP since Aβ decreased LTP magnitude to a similar extent (*p* > 0.05) in the absence (−47.84 ± 10.82%, n = 6) or presence of DPCPX (−41.98 ± 11.35%, n = 6) (Figure 4B,C).

The selective A_2A_R antagonist SCH58261 (50 nM) did not alter the PPF ratio (n = 5; Figure 4D) and failed to prevent the impact of Aβ on PPF (n = 5; Figure 4D) since this is essentially an A_1_R-regulated process [51]. In contrast, SCH58261 decreased LTP magnitude (62.60 ± 10.14% over baseline in the absence and 1.42 ± 2.09% in the presence of SCH58261; n = 5; *p* < 0.05; one-way ANOVA followed by Tukey’s multiple comparison post hoc test) and reverted the impact of Aβ on LTP (42.45 ± 9.17% over baseline in the presence of SCH58261 + Aβ; n = 5; *p* > 0.05 vs. SCH58261 alone) (Figure 4E,F). Likewise, the in vivo administration of Aβ also reduced LTP magnitude, an effect also reverted by the administration of SCH58261 to hippocampal slices from Aβ-treated mice (Figure 4G,H). This reinforces the ability of our optimized in vitro model of slices exposed to Aβ to mimic the effects of in vivo Aβ treatment on synaptic plasticity.

### 3.4. A_2A_R in Forebrain Neurons Are Involved in Aβ-Induced LTP Deficits

**Figure 5:**
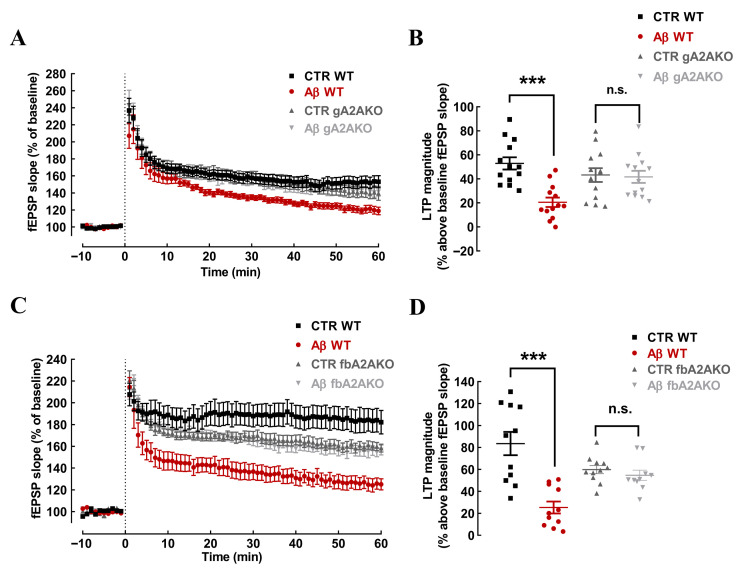
The impact of in vitro acute exposure to Aβ_1–42_ on mouse hippocampal LTP requires neuronal A_2A_R. Hippocampal slices from global A_2A_R knockout mice (gA_2A_RKO; **A**,**B**) or from forebrain neuronal A_2A_R knockout mice (fbA_2A_RKO mice; (**C**,**D**)) displayed a significantly lower LTP magnitude when compared with slices from control WT mice. Notably, acute Aβ administration to slices attenuated LTP magnitude in WT mice, but was devoid of effects in both gA_2A_RKO (**A**,**B**) or fbA_2A_RKO (**C**,**D**). Data are mean ± S.E.M. of 13 (**A**,**B**) and 10–11 (**C**,**D**) experiments; *** *p* < 0.001 compared to control, n.s. *p* > 0.05 compared to CTR gA_2A_RKO (**B**) or CTR fbA_2A_RKO (**D**); one-way ANOVA followed by Tukey’s multiple comparison post hoc test.

We next took advantage of mice where A_2A_R were selectively and globally deleted (gA_2A_RKO), to confirm the involvement of A_2A_R in the control of Aβ-induced depression of hippocampal LTP. As shown in Figure 5A,B, Aβ decreased LTP magnitude by 32.35 ± 6.12% (n = 13; *p* < 0.001) in wild type (WT) mice, whereas it failed to modify LTP magnitude in gA_2A_RKO (difference in LTP magnitude of −1.53 ± 8.38%, n = 13; *p* > 0.05, repeated measures one-way ANOVA followed by post hoc Tukey’s multiple comparisons test) (Figure 5A,B).

To gauge the selective involvement of neuronal A_2A_R, we tested the effect of Aβ in hippocampal slices of mice with a selective deletion of A_2A_R in forebrain neurons (fbA_2A_RKO) [43]. Whereas Aβ decreased LTP magnitude in WT control slices (83.53 ± 10.66% vs. baseline in the absence and 25.28 v 5.49% in the presence of Aβ; n = 11; *p* < 0.001, one-way ANOVA followed by post hoc Tukey’s multiple comparisons test) (Figure 5C,D), Aβ did not modify LTP magnitude in slices from fbA_2A_RKO (59.79 ± 3.68% in the absence and 54.69 ± 4.62% in the presence of Aβ; n = 10–11; *p* > 0.05, one-way ANOVA followed by post hoc Tukey’s multiple comparisons test) (Figure 5C,D). These observations confirm the mandatory role of neuronal A_2A_R on Aβ-induced depression of hippocampal LTP.

### 3.5. CD73-Mediated Formation of Extracellular Adenosine Is Critical for Aβ-Induced Depression of LTP

Since A_2A_R-mediated control of LTP in different brain areas depends on CD73-mediated formation of extracellular adenosine [30,38,52], we tested the impact of a pharmacological inhibition and of a genetic elimination of CD73 on the Aβ-induced depression of hippocampal LTP.

**Figure 6:**
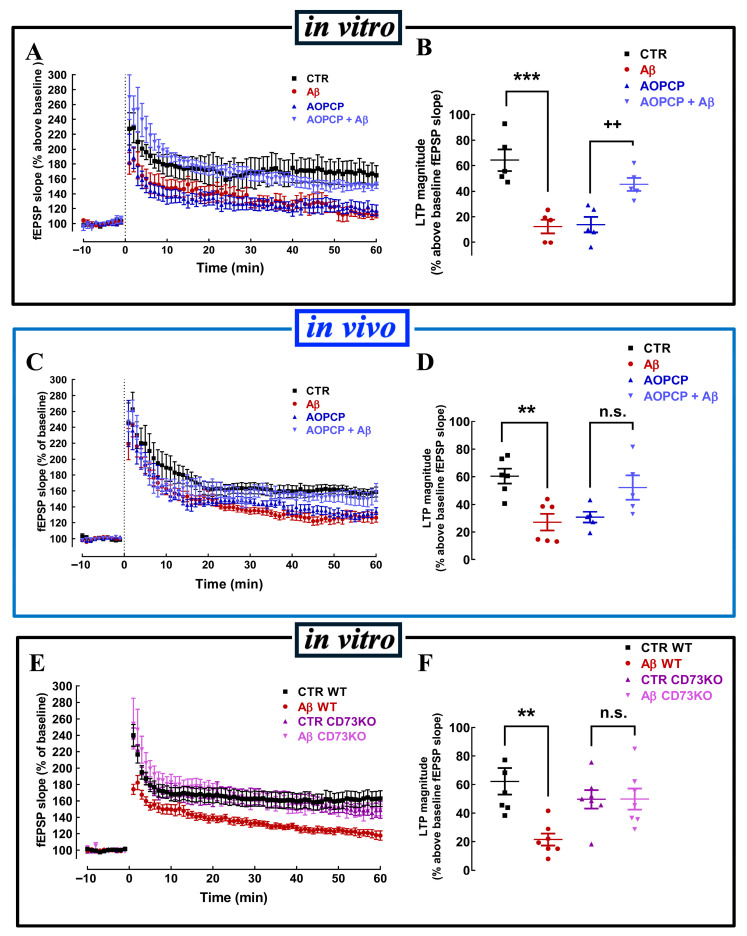
Aβ-induced deficits of hippocampal LTP depend on CD73-mediated formation of extracellular adenosine. Acute in vitro exposure of mouse hippocampal slices to Aβ decreased LTP magnitude, an effect that was not present upon a previous superfusion of the slices with the CD73 inhibitor α,β-methylene ADP (AOPCP, 100 μM, 20 min) (**A**,**B**), an effect that is identical to what is observed in hippocampal slices collected 14 days after in vivo icv injection of Aβ (**C**,**D**). This absence of impact of acute Aβ (50 nM, 40 min) on LTP was also replicated in hippocampal slices from CD73 knockout mice (CD73KO) (**E**,**F**). Data are mean ± S.E.M. of 5 (**A**,**B**), 5–6 (**B**,**C**), and 7 (**E**,**F**) experiments; ** *p* < 0.01, *** *p* < 0.001 compared to control, ++ *p* < 0.05 compared to AOPCP (**B**), n.s. *p* > 0.05 compared to AOPCP (**D**) or CTR CD73KO (**F**); one-way ANOVA followed by Tukey’s multiple comparison post hoc test.

In our optimized in vitro model, the CD73 inhibitor α,β-methylene ADP (AOPCP, 100 μM) mimicked the effects of SCH58261, decreasing LTP magnitude (64.31 ± 8.52% over baseline in the absence and 13.79 ± 6.05% in the presence of AOPCP; n = 5; *p* < 0.05; one-way ANOVA followed by Tukey’s multiple comparison post hoc test), and reverted the impact of Aβ on LTP (45.37 ± 5.06% over baseline in the presence of AOPCP + Aβ; n = 5; *p* > 0.05 *vs*. AOPCP alone) (Figure 6A,B). The effect of AOPCP on the in vitro effect of Aβ matched the in vivo effect of Aβ. Thus, in slices from mice exposed to intracerebroventricularly injected Aβ, the superfusion with AOPCP also reverted the effects of Aβ on LTP (Figure 6C,D). Likewise, in vitro application of Aβ also failed to alter LTP magnitude in slices from CD73KO mice (0.16 ± 9.98%; n = 7; *p* > 0.05), in contrast to the ability of Aβ to decreased LTP magnitude in WT mice (−40.84 ± 9.98%; n = 7; *p* < 0.01, one-way ANOVA followed by post hoc Tukey’s multiple comparisons test) (Figure 6E,F).

## 4. Discussion

The present study established an in vitro model of selective alterations of hippocampal long-term potentiation (LTP) upon acute exposure to β-amyloid peptide fragment 1_–_42 (Aβ), which mimics the impact of the in vivo intracerebroventricular administration of Aβ as a mouse model of early AD. This in vitro model affords superior experimental reproducibility, mechanistic resolution, and translational screening potential to identify drugs controlling Aβ-induced impairment of LTP. Indeed, the exploitation of this model allowed us to demonstrate that the Aβ-induced impairment of LTP occurs through a mechanism involving the selective overfunction of neuronal adenosine A_2A_ receptors (A_2A_R) via CD73-mediated formation of extracellular adenosine.

There is a remarkable consensus indicating that synaptic plasticity, namely LTP, is affected in different animal models of AD (reviewed in [53,54]), either based on the in vitro [46,47,48,49,50] or in vivo exposure to amyloid peptides (e.g., [55,56]), tau protein fragments (e.g., [57,58]), or different genetic AD models (e.g., [12,13,59]). Altered synaptic plasticity is an early process in AD, in accordance with the hypothesis that the onset of AD involves a synaptic dysfunction [6,7]. However, it is important to note that our data indicate that Aβ does not trigger an overt synaptotoxicity (which would be expected to curb all synaptic activity) but a rather selective alteration of synaptic adaptation. In fact, Aβ caused a consistent alteration of LTP magnitude without modifying basal synaptic transmission at hippocampal glutamatergic synapses, implying that Aβ is likely affecting particular mechanisms that are selectively engaged during plasticity rather than fundamental mechanisms of excitatory neurotransmission. The inhibitory impact of Aβ on LTP is observed both upon in vitro as well as upon in vivo exposure to Aβ, whereas there is a less consistent alteration of short-term plasticity. Thus, we observed a reduced PPF after acute in vitro Aβ exposure, and an unchanged PPF after in vivo Aβ injection. This likely results from compensatory presynaptic adaptations, differential engagement of glial or other neuromodulation systems, or differences in Aβ aggregation state and exposure kinetics in vivo rather than reflecting a presynaptic mechanism of action of Aβ, in agreement with different studies reporting different alterations of PPF upon exposure to Aβ [60,61,62] or in different mouse models of AD [12,59,63,64].

Several previous studies have reported that Aβ can bind, accumulate, and alter the function of synapses (reviewed in [53,65,66,67]). Thus, Aβ can affect mitochondria and the metabolic support of synapses (e.g., [68,69]), as well as glutamate receptors, namely NMDA receptors (e.g., [48,70]), and different transducing pathways involved in synaptic adaptation and silencing (e.g., [46,47,48,61,71,72]). Apart from direct effects in synapses, Aβ can also act in non-synaptic targets indirectly, formatting synaptic function such as depressing astrocytic-mediated glutamate uptake [73,74], microglia-associated neuroinflammation [18,75], the function of interneurons [76,77], and glymphatic and vascular functions [78,79]. These multiple effects of Aβ in different cellular compartments cast doubts on the mechanisms underlying the selective ability of Aβ to depress LTP without affecting basal synaptic transmission. The present study unveiled a key role of the overactivation of adenosine A_2A_ receptors (A_2A_R) in the Aβ-induced depression of hippocampal LTP. In fact, the removal of extracellular adenosine, impeding the formation of ATP-derived extracellular adenosine, or the pharmacological or genetic blockade of A_2A_R were sufficient to abrogate the ability of Aβ to inhibit hippocampal LTP. Notably, it is well-established that A_2A_R selectively control hippocampal LTP without affecting basal synaptic transmission or short-term plasticity [38,39,40]. This consists of a synapse-autonomous mechanism of increased ATP release from synapses with increased intensity of stimulation [80,81] and a conversion of this extracellular ATP into adenosine by a series of ecto-nucleotidases within the synapse [82], leading to an overactivation of A_2A_R in these synapses [30,52]. This scenario is fully consistent with the pivotal function of neuronal A_2A_R receptors to control memory deterioration [12,24,27,30,83], which reinforces the idea that the suppression of hippocampal plasticity by Aβ is primarily a synapse-autonomous phenomenon. This conclusion should not completely eliminate a potential astrocytic or microglial contribution to the extracellular adenosine pools [84,85] as well as a potential contribution of glial or vascular A_2A_R-mediated components in the Aβ-mediated impairment of hippocampal LTP [86,87].

In recent years, compelling evidence has accumulated pointing towards a crucial neuroprotective effect of adenosine signaling in several brain diseases (reviewed in [88]), including in the pathogenesis of AD [11,12,13,24,28,29,30,32]. The participation of adenosine and A_2A_R in the imbalance of LTP upon direct exposure to Aβ was evident when the receptor was blocked, either by the antagonist SCH58261 or through genetic elimination in a global A_2A_R knockout mouse. Likewise, in in vivo models of AD, the inhibition of A_2A_R function similarly prevents deficits of synaptic plasticity, which correlates with a reversion of memory impairment [12,13,24,28,30]. These findings are in notable agreement with the reported prophylactic benefits of coffee and of caffeine, the most consumed psychoactive drug worldwide, mainly acting as an adenosine receptor antagonist in the brain when consumed regularly and in moderate doses [37], to attenuate memory deterioration in AD [31,32,33,34,35,36]. Additionally, animal studies have revealed that A_2A_R overfunction is both strictly necessary [11,12,13,24,28,29,30,32] and actually sufficient to trigger memory impairments [24,83,89]. Overall, the evidence posits A_2A_R overfunction as a new target to interfere with AD.

A_2A_R overfunction in AD has been conceived as resulting from the increased density of A_2A_R in afflicted brain areas [11,12,13,24,28,29,30], in particular within synapses [11,12,13,30] but also in astrocytes [73,86], although memory dysfunction has largely been attributed to the dysregulation of neuronal A_2A_R [12,24,30,90]. The present study reinforces our previous contention that A_2A_R overfunction further results from an increased extracellular catabolism of ATP into adenosine via CD73/ecto-5′-nucleotidase. We previously observed that the genetic elimination of CD73 leads to a positive outcome in an early AD model [30], an effect also described in the control of fear memory [52]. Remarkably, the present data obtained in our neurophysiological model were consistent with those previous observations, suggesting that Aβ-induced changes in LTP involve a coordinated dysfunction of an integrated axis of the purinergic system, consisting of increased ATP release, increased CD73-mediated formation of extracellular adenosine, and overfunction of neuronal A_2A_R. This conclusion prompts further studies to address the impact of Aβ on ATP release from nerve terminals and to test if the control of the exacerbation of this ‘danger signaling’ mechanism [91] is a viable alternative to counteract the negative impact of Aβ on LTP.

Overall, our results show that acute administration of Aβ in mouse hippocampal slices dampens LTP, mimicking in vivo models of AD, through a mechanism involving an overfunction of the CD73-adenosine-A_2A_R axis, as previously concluded in in vivo models of AD [30]. This novel neurophysiological model conveys a simplified yet robust approach with significant advantages in terms of time and cost, to screen and select new drugs controlling the impact of Aβ in hippocampal LTP as novel candidate drugs to manage AD.

## Data Availability

The original contributions presented in this study are included in the article. Further inquiries can be directed to the corresponding author.
